# ABO and RhD blood group antigen frequencies in patients and blood donors: Implications for the U.S. blood supply

**DOI:** 10.1371/journal.pone.0356683

**Published:** 2026-08-25

**Authors:** Elizabeth S. Allen, Sara Bakhtary, Sarah E. Barnhard, Roksolana Demianets, Gagan Mathur, Catherine Mazzei, Zhen Mei, Suzanne L. Wheeler, Khalda A. Ibrahim, on behalf of the University of California Transfusion Medicine Physicians

**Affiliations:** 1 Department of Pathology, University of California San Diego, La Jolla, California, United States of America; 2 Department of Laboratory Medicine, University of California San Francisco, San Francisco, California, United States of America; 3 Department of Pathology, University of California Davis, Sacramento, California, United States of America; 4 Department of Pathology and Laboratory Medicine, University of California Irvine, Orange, California, United States of America; 5 American Red Cross, San Leandro, California, United States of America; 6 Department of Pathology and Laboratory Medicine, University of California Los Angeles, Los Angeles, California, United States of America; Roswell Park Cancer Institute, UNITED STATES OF AMERICA

## Abstract

**Background:**

ABO/Rh blood groups play a pivotal role in the practice of transfusion medicine. To our knowledge, there have been no large studies reporting these blood group antigen frequencies in the United States (US) since 2004, and the most recent large study only reported antigen frequencies for donors, not patients. We sought to determine and compare the ABO/RhD antigen frequencies in patient and donor populations.

**Methods:**

In this multi-center retrospective cohort study, five California academic medical centers and one national blood center reviewed blood group and demographic data from patients, California blood donors, and US blood donors who underwent ABO/RhD typing during a 5-year period. Data were analyzed to characterize each population and to compare the patient and donor populations.

**Results:**

ABO/RhD typing for 467,748 patients, 719,211 California blood donors, and 7,449,831 US blood donors were included in this study. Among patients, California donors, and US donors, group O was represented by 46.2%, 50.8%, and 48.8% of the population, respectively; group A 35.8%, 32.1%, and 36.0%; group B 14.1%, 13.0%, and 11.3%; and group AB 3.9%, 4.1%, and 3.9%. Among patients, California donors, and US donors, RhD-positive individuals represented 90.0%, 88.4%, and 83.0% of the population. Patient ABO/RhD frequencies varied significantly by site, age, and sex, and differed from the ABO/RhD frequencies of California and US donors.

**Conclusions:**

This study reports ABO/RhD frequencies in a large cohort of California patients, California blood donors, and US blood donors, which may inform donor recruitment, inventory management, and policy development. Patient and donor ABO/RhD frequencies differ significantly, indicating potential misalignment that may be consequential and warrants investigation.

## Introduction

The ABO and RhD blood group antigens (hereafter referred to as ‘antigens’) are clinically significant and foundational to transfusion medicine and immunohematology. Blood donors and patients are routinely typed for ABO and RhD, and ABO compatibility is an essential component of patient safety for all red blood cell (RBC) transfusions [1].

Consequently, ABO/RhD blood grouping shapes policy and drives inventory management for both blood centers and hospital transfusion services. Demand for universal donor products such as O, RhD-negative RBCs and AB plasma—for emergencies and specific populations (e.g., neonates, patients undergoing hematopoietic stem cell transplantation)—exceeds their true population frequencies [2,3], and during shortages, blood centers have curtailed group O RBC availability [4]. Although only about 7% of United States (US) blood donors are group O, RhD-negative, these units account for about 10% of distributed components [2,3]. Experts now recommend using type-specific RBC units when possible [5] and reserving O, RhD-negative units for defined situations [5–8], while encouraging broader use of O, RhD-positive units to preserve the limited O, RhD-negative stock [9–12]. Judicious stewardship of blood products by ABO/RhD group is essential to maintain an adequate blood supply and optimize transfusion service operations.

Navigating these challenges to facilitate a sustainable blood supply requires accurate information about patient and donor ABO/RhD frequencies, which is surprisingly limited. First, multiple widely-used reference texts report ABO/RhD frequencies by race or ethnicity [13–15], which fails to inform about overall ABO/RhD frequencies among institutional or regional populations. Moreover, as global migration increases [16], ABO/RhD frequencies may be shifting at local, regional, and national levels. Yet they are routinely reported in key texts with missing [1] or out-of-date source data [17,18], with the most recent reference being a single 2004 study of US blood donors [19]. To our knowledge, US donor ABO/RhD frequencies have not been reported since, and large population data characterizing *patient* ABO/RhD frequencies may be even older. Regular analysis and reporting of prevailing ABO/RhD frequencies are critical to keeping transfusion practice current with population changes.

In short, most existing data on antigen frequencies are not current and/or are reported based on outdated racial and ethnic groupings, and thus do not reflect current populations or provide a comprehensive picture. In this study, we determined the ABO and RhD antigen frequencies in a large cohort of patients typed at the five University of California medical centers, aiming to characterize this patient population and to compare its ABO/RhD distributions with those of blood donors typed at the American Red Cross in California and across the US overall.

## Methods

In this retrospective, multi-center study, five University of California (UC) medical centers (S1 Table) collected blood type and demographic data for all patients who underwent blood typing over a five-year period (March 1, 2018 – February 28, 2023). The American Red Cross (ARC), which collects blood in 44 states through mobile blood drives and nearly 200 permanent donation centers and supplies about 40% of US blood and blood components [20,21], collected similar data on donors during the same time period. The study was approved by the institutional review boards at all five medical centers.

### Data queries

Between July 15, 2023 and April 15, 2024, each medical center queried its electronic health record system (Epic Systems Corporation, Verona, WI) or laboratory information system (SafeTrace Tx, Haemonetics Corporation, Boston, MA; SCC Soft Computer, Clearwater, FL; Sunquest Information Systems, Tucson, AZ; WellSky, Overland Park, KS) to determine the blood types and demographic data—in aggregate—of patients tested within the designated five-year timeframe. ARC queried its blood establishment computer system, eProgresa (Mak System, Skopje, North Macedonia), on September 24, 2024 to determine the blood types of individuals who donated blood during the designated timeframe. Data sets were based on unique individuals at each facility rather than number of tests or donations. Personally identifiable information was not included in the accessed data at any site.

### Reported race

We analyze data on race based on the following categories defined by the US Office of Management and Budget (OMB) that were in effect until March 2024: [22] American Indian or Alaska Native; Asian; Black or African American; Native Hawaiian or Other Pacific Islander; and White. Race data for US and California populations were obtained from the 2020 US Census [23]. In addition to the OMB categories, the US Census Bureau allows a response of “Other race or multiple races.”

Race was self-reported by patients and donors. In addition to the categories defined by OMB, patients at UC medical centers could also select responses such as “Other/Mixed,” “Some other race,” “Two or more races,” “Not reported/unknown,” “Declined/unknown,” and “None of the above.” Donors at ARC had additional response options including “Mix,” “Other,” and “Prefer not to answer.”

The response options used by the study institutions were matched by two authors (KAI, ESA) to the most similar OMB category. If there was no corresponding OMB category, the response was classified as “Other.” If no self-reported race was available, the response was categorized as “Not Reported/Unknown.” (See S2 Table)

### Blood group antigen typing methods

Patients were typed by automated column agglutination technique (Grifols Diagnostics Solutions, Emeryville, CA, and Ortho Clinical Diagnostics, Raritan, NJ) or by automated solid-phase technique (Immucor, Norcross, GA), with reflex to tube testing as needed. Donors were typed by automated solid-phase technique (analyzer: Beckman Coulter, Brea, CA; reagents: DIAGAST, Loos, France). Results were reported as “O, RhD-positive,” “A, RhD-negative,” etc. Patients and donors with indeterminate blood typing were excluded.

### Statistical analysis

Data were aggregated and descriptive statistics were derived using Microsoft Excel (Microsoft Corporation, Redmond, WA), R 4.3.2 (R Foundation for Statistical Computing, Vienna, Austria), and RStudio version 2024.4.2.764 (Posit Software, PBC, Boston, MA). Statistical tests of population differences included the Pearson’s chi-squared test and were performed using R and RStudio.

## Results

In total, 467,748 patients across five UC medical centers, 719,211 blood donors across the state of California, and 7,449,831 blood donors (representing about 2.1% of the estimated US population) across the US were analyzed. First, UC patient demographics were compared to the populations of California and the US as reported in the US Census [23]. Next, UC patient blood types were analyzed across sites and by age and sex. Finally, UC patient blood types were compared to those of ARC donors from California and throughout the US.

### Demographic analysis by self-reported race

**Fig 1:**
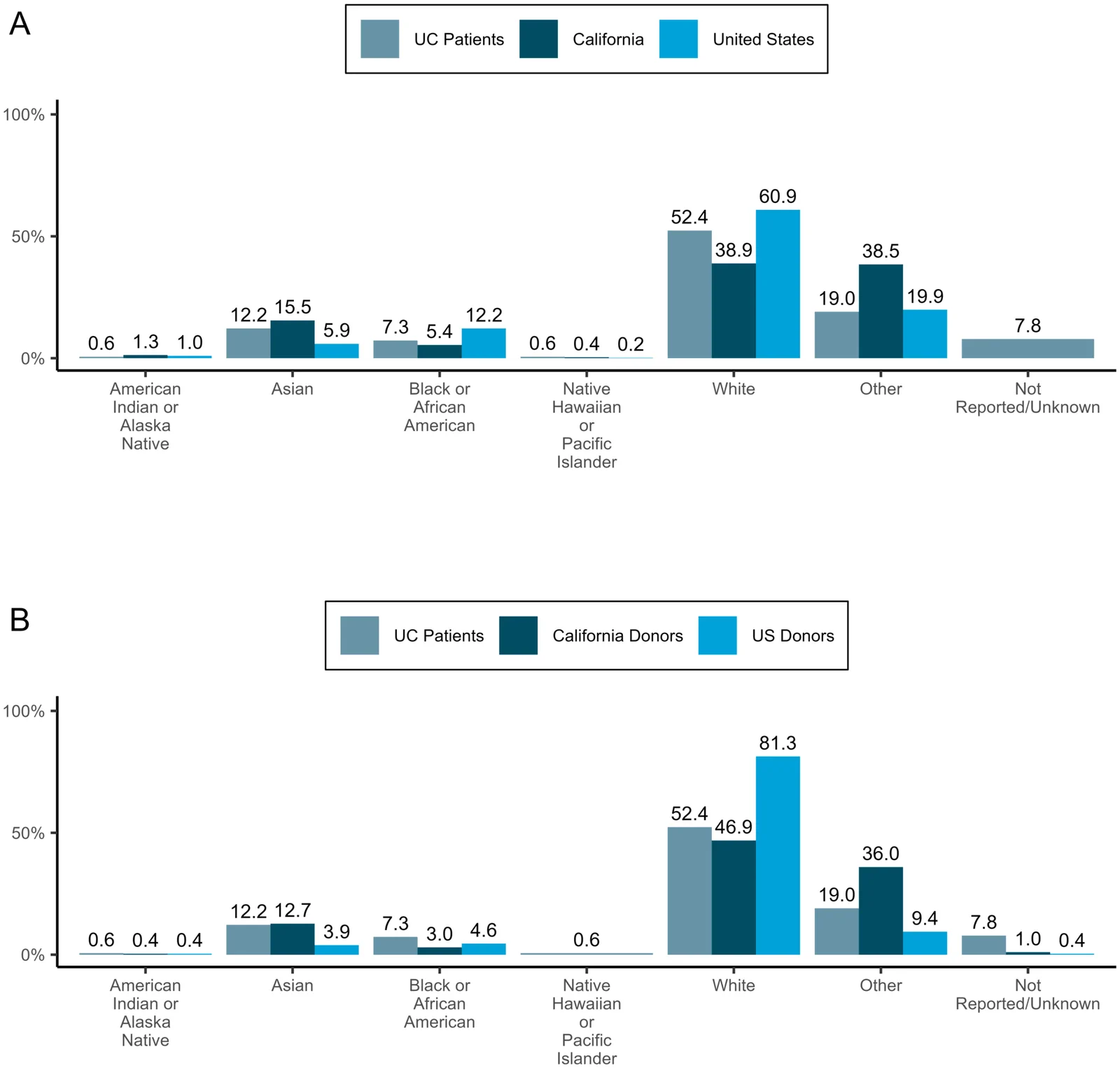
UC patient demographics compared to those of the general population and blood donors. Races reported among UC patients, compared to (A) the California population and the US population (as reported by the US Census), and (B) the California blood donor population and the US blood donor population (as reported by the American Red Cross).

Comparison of UC patient data to US census data revealed differences in distributions by race (Fig 1), although analysis was limited by incomplete data. Because patients at some UC sites could select more than one category for race, the denominator for analysis by self-reported race is 475,777. The UC patient and California populations demonstrated higher proportions of Asian individuals (12.2% and 15.5%) compared to the US population overall (5.9%). Also noted were lower proportions of individuals identifying as Black or African American (7.3% and 5.4%) and White (52.4% and 38.9%) compared to the US population (Black or African American 12.2%; White 60.9%). Full statistical analysis (including subgroup analysis) was not feasible due to missing data and misaligned terminology. For example, within the UC patient population, significant proportions of patients identified as “Other” (19.0%) or had race reported as “Not Reported/Unknown” (7.8%).

Comparison of UC patients to blood donors (both statewide and nationwide) revealed similar differences (Fig 1), although analysis was again limited by incomplete data. The UC patient and California blood donor populations demonstrated higher proportions of individuals identifying as Asian (12.2% and 12.7%) compared to the overall US blood donor population (3.9%). Likewise, lower proportions of individuals identified as White (52.4% and 46.9%, respectively) compared to the US blood donor population (81.3%). In contrast to national-level Census data on the US population, the proportion of UC patients identifying as Black or African American (7.3%) was higher than that of California blood donors (3.0%) and of US blood donors (4.6%). Full statistical analysis (including subgroup analysis) was not feasible due to missing data and misaligned terminology. Of note, data on Hispanic ethnicity were not available at all of the UC medical centers.

### ABO and RhD antigen frequencies among UC patients

Among UC patients, blood groups O, A, B, and AB represented 46.2%, 35.8%, 14.1%, and 3.9% of the population, respectively. The proportions of RhD-positive and RhD-negative patients were 90.0% and 10.0%, respectively (Table 1).

**Table 1:** Blood group antigen frequencies among UC patients, American Red Cross California blood donors, and American Red Cross national blood donors.

	O, RhD+	O, RhD-		A, RhD+	A, RhD-	B, RhD+	B, RhD-	AB, RhD+	AB, RhD-	Total
UC Patients, n (%)	195,369 (41.8)	20,697 (4.4)		148,705 (31.8)	18,820 (4.0)	60,309 (12.9)	5,414 (1.2)	16,583 (3.5)	1,851 (0.4)	467,748 (100)
CA Donors, n (%)	321,380 (44.7)	44,259 (6.2)		203,610 (28.3)	27,412 (3.8)	84,677 (11.8)	8,641 (1.2)	26,113 (3.6)	3,119 (0.4)	719,211 (100)
US Donors, n (%)	2,982,684 (40.0)	654,486 (8.8)		2,239,874 (30.1)	439,509 (5.9)	715,099 (9.6)	125,279 (1.7)	247,963 (3.3)	44,937 (0.6)	7,449,831 (100)
	**O**		**A**	**B**	**AB**	**RhD+**	**RhD-**	**Total**	*p* value*	
UC Patients, n (%)	216,066 (46.2)		167,525 (35.8)	65,723 (14.1)	18,434 (3.9)	420,966 (90.0)	46,782 (10.0)	467,748 (100)	comparator	
CA Donors, n (%)	365,639 (50.8)		231,022 (32.1)	93,318 (13.0)	29,232 (4.1)	635,780 (88.4)	83,431 (11.6)	719,211 (100)	ABO: <0.0001 RhD: <0.0001	
US Donors, n (%)	3,637,170 (48.8)		2,679,383 (36.0)	840,378 (11.3)	292,900 (3.9)	6,185,620 (83.0)	1,264,211 (17.0)	7,449,831 (100)	ABO: <0.0001 RhD: <0.0001	

CA—California; UC—University of California.

* Based on 2x4 (ABO) or 2x2 (RhD) chi square test comparing the UC patient population to the listed donor population.

Analysis by medical center revealed differences between sites. The proportion of group O patients ranged from 44.2% at UC San Francisco to 49.4% at UC Irvine; the proportion of group A patients ranged from 30.2% at UC Irvine to 37.6% at UC Davis; and the proportion of group B patients ranged from 12.7% at UC San Diego to 16.7% at UC Irvine. The frequencies of ABO groups among sites differed significantly [X^2^ (df = 12, N = 467,748) = 1696, *p* < 0.0001]. The proportion of RhD-positive patients ranged from 88.9% at UC Davis to 92.2% at UC Irvine. The frequencies of RhD-positive and -negative patients also differed significantly among sites [X^2^ (df = 4, N = 467,748) = 475, *p* < 0.0001].

**Fig 2:**
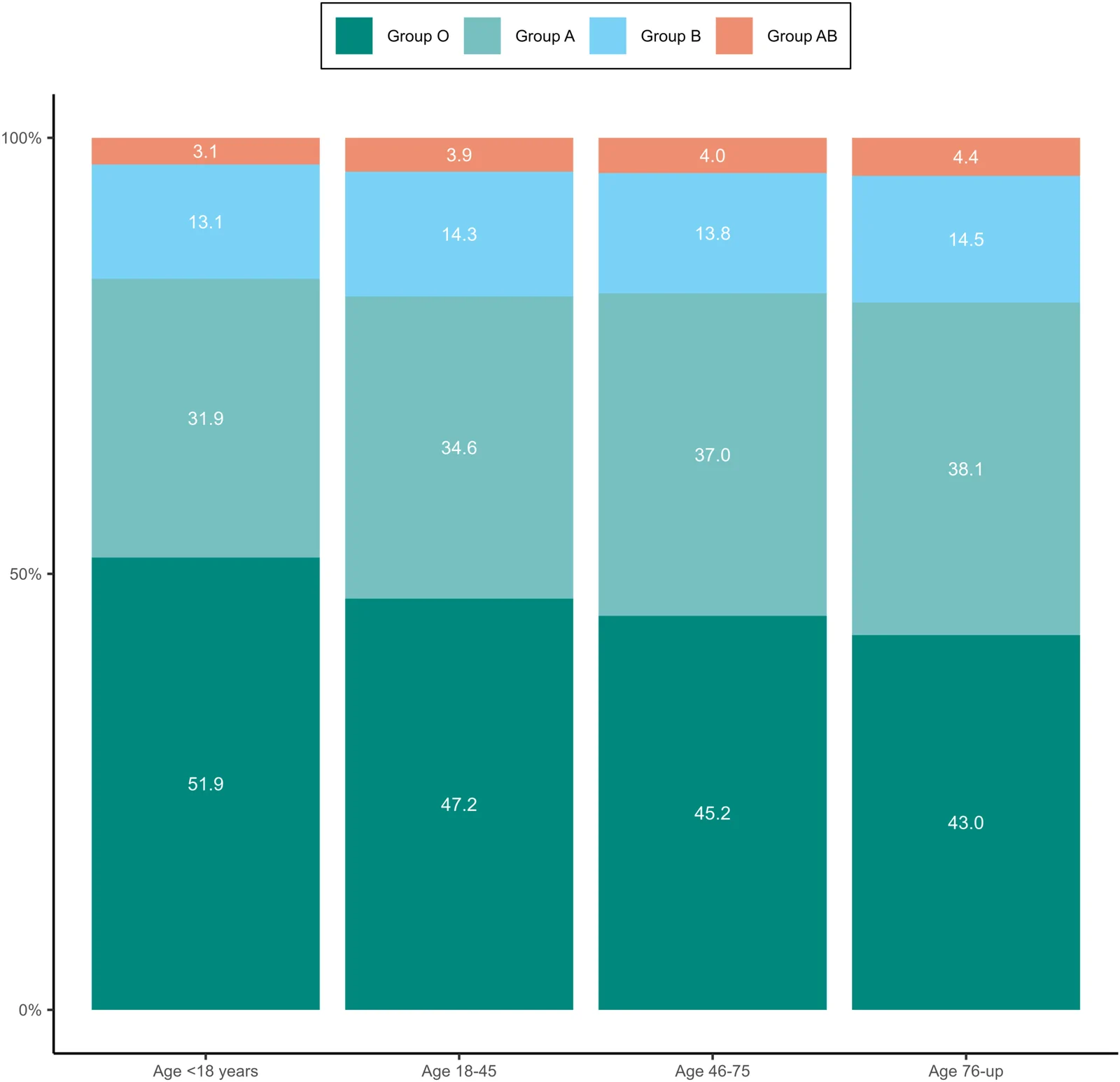
ABO antigen frequencies of UC patients stratified by age group. Comparison of ABO antigen frequencies among UC patients by age.

Analysis by age also revealed differences (Table 2, Fig 2). Patients were stratified into four age groups: under 18 years (n = 32,090; 6.9%), 18–45 years (n = 191,542; 40.9%), 46–75 years (n = 189,814; 40.6%), and 76 years or more (n = 54,302; 11.6%). The proportion of group O patients correlated inversely with age, ranging from 43.0% in patients 76 years and older to 51.9% in patients under 18 years. The proportion of group A patients correlated directly with age, ranging from 31.9% in patients under 18 years up to 38.1% in patients 76 years and older. Similarly, the proportion of group AB patients ranged from 3.1% in patients under 18 years to 4.4% in patients 76 years and older. The proportion of group B patients ranged from 14.5% in patients 76 years and older to 13.1% in patients under 18 years. The frequencies of ABO types among age groups differed significantly [X^2^ (df = 9, N = 467,748) = 928, *p* < 0.0001]. In light of the limited number of patients less than 18 years, additional analysis comparing ABO frequencies among only the three older age groups was performed and also showed significant differences [X^2^ (df = 6, N = 435,658) = 455, *p* < 0.0001].

**Table 2:** Blood group antigen frequencies among UC patients, stratified by age range and sex.

	O, RhD+	O, RhD-	A, RhD+	A, RhD-	B, RhD+	B, RhD-	AB, RhD+	AB, RhD-	Total
**Age, years (n, %)**									
< 18	15,239 (47.5)	1,406 (4.4)	9,160 (28.5)	1,090 (3.4)	3,847 (12.0)	364 (1.1)	883 (2.8)	101 (0.3)	32,090
18-45	82,621 (43.1)	7,712 (4.0)	59,556 (31.1)	6,805 (3.6)	25,411 (13.3)	2,014 (1.1)	6,791 (3.5)	632 (0.3)	191,542
46-75	76,790 (40.5)	8,962 (4.7)	61,857 (32.6)	8,348 (4.4)	23,876 (12.6)	2,320 (1.2)	6,808 (3.6)	853 (0.4)	189,814
76+	20,719 (38.2)	2,617 (4.8)	18,132 (33.4)	2,577 (4.7)	7,175 (13.2)	716 (1.3)	2,101 (3.9)	265 (0.5)	54,302
Sex* (n, %)									
Male	81,984 (41.7)	8,953 (4.6)	62,779 (32.0)	8,215 (4.2)	24,470 (12.5)	2,315 (1.2)	6,842 (3.5)	826 (0.4)	196,384
Female	113,310 (41.8)	11,735 (4.3)	85,852 (31.7)	10,597 (3.9)	35,821 (13.2)	3,101 (1.1)	9,733 (3.6)	1,025 (0.4)	271,174

* Additionally, 193 individuals (0.04%) reported a sex of genderqueer, non-binary, transgender, “None of the above,” or chose not to report a sex. From the remaining population, analysis by sex shows an overcounting of 3 individuals (< 0.01% of the UC patient population), attributed to individuals who selected more than one category. Percentages do not sum to 100 due to rounding.

The proportion of RhD-positive patients correlated inversely with age, ranging from 88.6% in patients 76 years and older to 90.8% in patients under 18 years. The proportions of RhD-positive and RhD-negative patients among age groups also differed significantly [X^2^ (df = 3, N = 467,748) = 497, *p* < 0.0001]. In light of the limited number of patients less than 18 years, additional analysis comparing RhD frequencies only in the three older age groups was performed and also showed significant differences [X^2^ (df = 2, N = 435,658) = 471, *p* < 0.0001].

Analysis by sex also demonstrated differences (Table 2). Of note, 193 individuals (0.04% of the patient population) reported neither male nor female sex: 38 individuals reported their sex as genderqueer, non-binary, or transgender; 20 individuals reported “None of the above”; and 135 individuals did not report a sex. Because the expected values for these groups were very low, application of Pearson’s chi-squared test for ABO and RhD distributions was restricted to patients reporting sex as male or female (N = 467,558). The proportions of group O and group AB patients were nearly equal in males (O 46.3%, AB 3.9%) and females (O 46.1%, AB 4.0%). The proportion of group A patients was slightly higher in males than in females (36.2% vs. 35.6%) and group B patients was slightly lower (13.6% vs. 14.4%). Whether the male and female populations showed similar age distributions could not be assessed from available data. The frequencies of ABO types between males and females differed significantly [X^2^ (df = 3, N = 467,558) = 54, *p* < 0.0001]. The proportions of RhD-positive patients among males and females, 89.7% and 90.2%, also differed significantly [X^2^ (df = 1, N = 467,558) = 43, *p* < 0.0001].

### ABO and RhD antigen frequencies of UC patients compared to California blood donors

**Fig 3:**
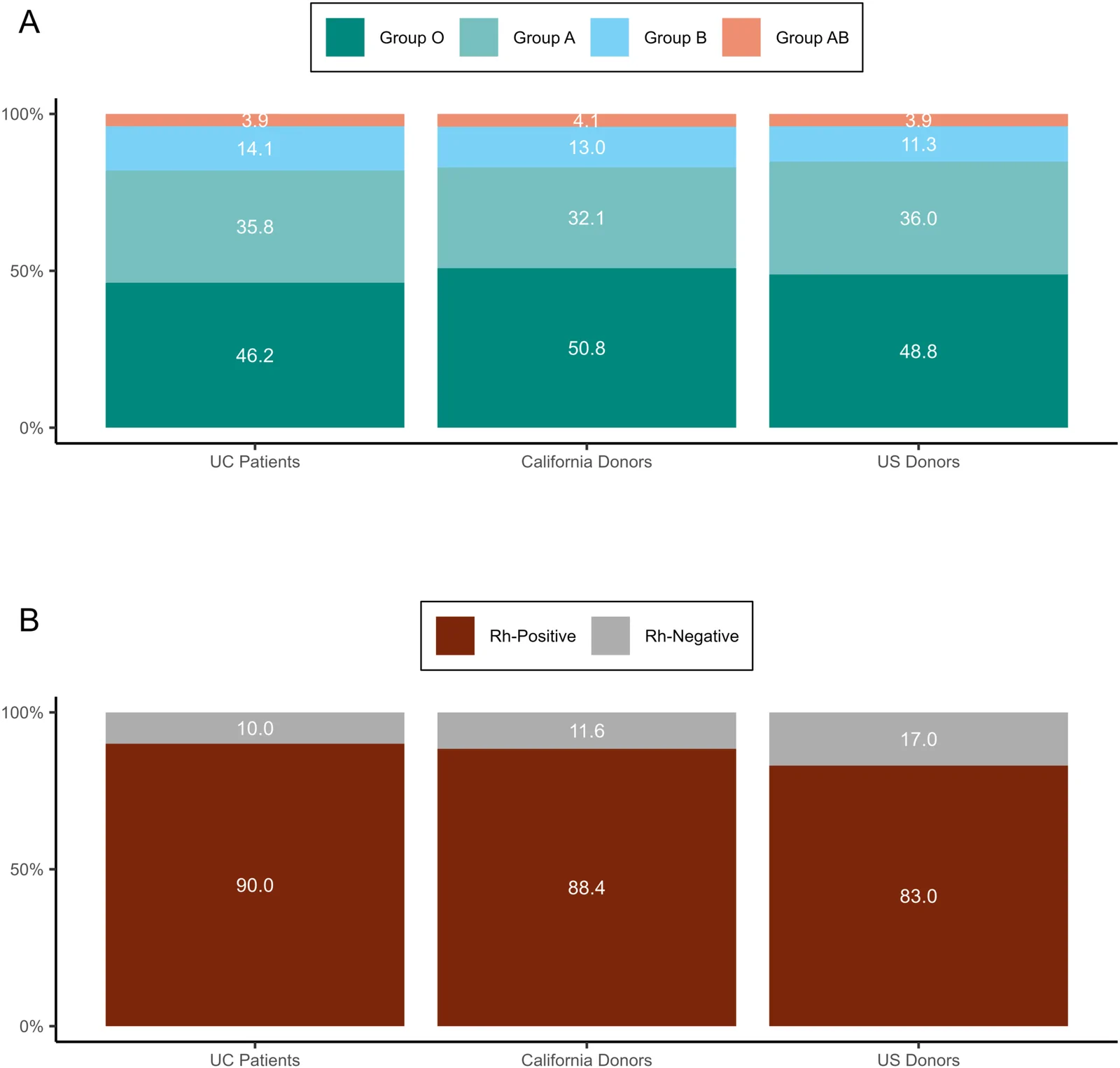
ABO and RhD antigen frequencies of UC patients compared to those of California and US blood donors. Comparison of (A) ABO and (B) RhD antigen frequencies among UC patients, US blood donors, and California blood donors at the American Red Cross.

Compared to the California blood donor population, UC patients had a smaller fraction of group O (46.2% vs. 50.8%) and a larger fraction of group B (14.1% vs. 13.0%) and RhD-positive (90.0% vs. 88.4%) individuals (Table 1). Among UC patients, the frequencies of ABO types and the proportion of RhD-positive patients were significantly different from those of the California blood donor population [ABO: X^2^ (df = 3, N = 467,748) = 2656, *p* < 0.0001; RhD: X^2^ (df = 1, N = 467,748) = 742, *p* < 0.0001] (Fig 3).

### ABO and RhD antigen frequencies of UC patients compared to US blood donors

Compared to the US blood donor population, UC patients had a smaller fraction of group O (46.2% vs. 48.8%) and a larger fraction of group B (14.1% vs. 11.3%) and RhD-positive (90.0% vs. 83.0%) individuals (Table 1). Among UC patients, the frequencies of ABO types and the proportion of RhD-positive patients were significantly different from those of the US blood donor population [ABO: X^2^ (df = 3, N = 467,748) =3580, *p* < 0.0001; RhD: X^2^ (df = 1, N = 467,748) = 15466, *p* < 0.0001] (Fig 3).

## Discussion

This study examined the demographic characteristics and ABO/RhD antigen frequencies of 467,748 patients across an academic health system in California, and comparisons to 719,211 California blood donors and 7,449,831 US blood donors revealed statistically significant differences among these populations. At a time when the population is changing, these data provide updated antigen frequencies for both patient and donor populations. Moreover, recent blood shortages have demonstrated that astute management and judicious product allocation are important to maintaining a stable blood supply in the US. Accurate data about ABO/RhD frequencies of patients and donors is an essential first step in creating a sustainable blood supply.

Surprisingly, there has been a dearth of up-to-date, readily accessible data describing ABO/RhD antigen frequencies in the US population. We reviewed publications from America’s Blood Centers [17] and the Association for the Advancement of Blood and Biotherapies (AABB) [18], both of which rely on data from a single 2004 study of blood donors [19]. Arguably, demographics within the US population have shifted considerably in the intervening 20 years [24–26]. This study fills a critical gap by providing current, state-level statistics on ABO/RhD antigen frequencies among patients, and similar state- and national-level statistics for blood donors. We are not aware of any other recent US studies that have provided ABO/RhD antigen frequency data for either patients or donors.

Historically, ABO and RhD antigen frequencies are often reported by race or ethnic group, which can be misleading and may not accurately characterize the population as a whole [27]. In fact, many individuals defy categorization: in this study, more than 25% of UC patients reported their race as “Other” or “Not reported/Unknown,” precluding further analysis. Furthermore, reporting by race does not necessarily provide information that translates to local, regional, or institutional populations. In this study, the inclusion of individuals who did not fit into existing race categories presents a more accurate picture (S3 Table). Additionally, reporting frequencies in aggregate rather than solely by race provides a robust overview of large and diverse groups of patients and donors.

Notably, we found a higher proportion of patients than expected were group B (14.1%) or RhD-positive (90.0%), a phenomenon that we hypothesize is multifactorial and may carry implications for national inventory management. It may be related to a larger proportion of patients of Asian or African descent, or to the types of disease specialization at these facilities. Moreover, since the transfusion of ABO-mismatched platelets may cause transfusion reactions [28–33] and/or impact efficacy [34,35], providing ABO-identical platelets could become a priority in national inventory management strategy. More broadly, the higher prevalence of group B individuals illustrates the importance of characterizing one’s own local population for optimal transfusion service operations. Reference material providing ABO/RhD frequencies may not accurately reflect the population served by a specific institution or region. As such, we suggest institutions characterize their own local ABO/RhD frequencies for maximum utility.

We observed statistically significant differences in the ABO/RhD antigen frequencies between patients and blood donors, which has ramifications throughout transfusion medicine. While some misalignment is expected because donor antigen frequencies are skewed towards recruitment goals, there are inherent limitations to this approach. For example, group O individuals represent 50.8% of California donors, but just 46.2% of UC patients, a difference of 4.6%. While their RBC units (especially O, RhD-negative) are sought by blood centers for their necessity in emergent transfusion situations, recent shortages have raised questions about whether these donors are exhausted, which has forced a re-evaluation of the widespread use of group O RBC units. Blood centers now promote increased provision of type-specific units, a cornerstone of inventory management [2,5–8] which sometimes has medical benefits [36]. Transfusion services have been assessing their practices [37–40] and implementing more thoughtful policies [11,12,39]. The findings in this study suggest additional, as yet untapped approaches. For example, institutions with higher proportions of group B patients may consider accepting more group B units, which could reduce overreliance on group O donors. Similarly, knowing the prevalence of RhD-negative patients may help transfusion services create policies for the optimal use of O, RhD-negative units, potentially reducing demand. In our data, 90% of patients were RhD-positive compared to 83% of US donors, a difference of 7%, suggesting there may be relative underrepresentation of Rh-positive units compared to patient demand. These findings support ongoing efforts to preferentially use type-specific blood products when clinically appropriate, to reconsider inventory distribution strategies that rely heavily on universal donor units, and to consider recruitment plans that better align donor demographics with patient needs. In summary, quantifying the incongruence between patient and donor blood type frequencies provides a framework to optimize donor recruitment, product distribution, and utilization practices to create a more sustainable blood supply.

Limitations of this study include the fact that UC patients are only a subset of Californian patients and may not be representative of larger groups. In particular, the UC academic medical centers may disproportionately serve groups with greater access to specialized tertiary care. Additionally, the study examines patients who underwent blood typing, which may not be representative of all patients in the UC Health system or the subpopulation who receive transfusions. Certain types of patients may be more or less likely to have blood typing performed (e.g., obstetric patients) and thus may be over- or under-represented. Nevertheless, those undergoing blood typing are likely a reasonable proxy for patients who are expected to receive transfusions and who are therefore of interest to transfusion services. An additional limitation is that age and sex data were not available for the blood donor population. In the US, blood donors must meet minimum age requirements (typically 16–17 years with parental consent, depending on location), and donor populations generally comprise healthy individuals within selected adult age ranges [41]. Since observed ABO and RhD frequencies among *patients* varied by both age and sex, demographic composition may influence observed blood group antigen distributions. Consequently, the differences between patient and donor populations may reflect differences in age and sex distributions rather than solely differences between the populations.

Finally, it was not possible to determine whether any patients had undergone blood typing at multiple UC medical centers, and therefore it was impossible to remove potential duplicate cases. However, by limiting the study period to 5 years we believe that we reduced the likelihood of patients being represented in multiple institutions’ data.

In conclusion, this study assessed the ABO/RhD antigen frequencies across a population of 467,748 patients at five California-based academic medical centers, 719,211 blood donors in California, and 7,449,831 blood donors across the US. Among patients, antigen frequencies varied significantly by site, age, and sex, highlighting the importance of characterizing frequencies within local populations. The ABO/RhD frequencies observed for patients also differed significantly from frequencies observed for both California and US blood donors. Patients demonstrated higher proportions of group B and RhD-positive individuals compared to the state and national donor pools. Blood centers typically recruit to prioritize group O, RhD-negative units to support emergency transfusion needs and other special populations. Nevertheless, the potential misalignment between ABO/RhD frequencies of blood donors and transfusion recipients suggests both challenges and opportunities. Future studies incorporating age- and sex-stratified analyses of blood donors may clarify the underlying etiology of the observed differences and could help refine donor recruitment strategies to better align the donor pool with patient transfusion needs. Accurate characterization of the distribution of ABO/RhD antigens in diverse patient and donor populations may aid in donor recruitment, inventory planning, transfusion service operations, and clinical decision-making, and ultimately reflect a significant opportunity to create a more sustainable blood supply.

## Supporting information

S1 Table Characteristics of the five UC medical centers. (DOCX)

S2 Table Harmonized terminology for self-reported race. (DOCX)

S3 Table Blood group antigen frequencies among California blood donors and US blood donors at the American Red Cross by race. (DOCX)

## References

[1] Cohn CS, Delaney M, Johnson ST, Katz LM, Schwartz J. ABO and Other Carbohydrate Blood Group Systems. Technical Manual. 21st ed. AABB. 2023. p. 303–31.

[2] Baker SA. Empower Group O Care: An Initiative of the American Red Cross. https://redcrossplus.blog/2025/03/11/empower-group-o-care-an-initiative-of-the-american-red-cross/

[3] Kracalik I, Sapiano MRP, Wild RC, Chavez Ortiz J, Stewart P, Berger JJ, et al. Supplemental findings of the 2021 National Blood Collection and Utilization Survey. Transfusion. 2023;63 Suppl 4(Suppl 4):S19–42. doi: 10.1111/trf.17509 37702255 PMC10783319

[4] O’Brien KL, Mohammed M, Uhl L. Management of a Hospital Transfusion Service During a Nationwide Blood Product Shortage. Arch Pathol Lab Med. 2018;142(7):779–81. doi: 10.5858/arpa.2017-0483-LE 29939778

[5] Murphy M, BenAvram D. Recommendations on the use of group O red blood cells.

[6] Gammon RR, Rosenbaum L, Cooke R, Friedman M, Rockwood L, Nichols T, et al. Maintaining adequate donations and a sustainable blood supply: Lessons learned. Transfusion. 2021;61(1):294–302. doi: 10.1111/trf.16145 33206404 PMC7753343

[7] Yazer MH, Díaz-Valdés JR, Triulzi DJ, Cap AP. Wider perspectives: It’s a changing world-The use of ABO-incompatible plasma for resuscitating massively bleeding patients. Br J Haematol. 2023;200(3):291–6. doi: 10.1111/bjh.18460 36134727

[8] AABB. Standards for blood banks and transfusion services. 34th ed. AABB Press. 2024.

[9] Selleng K, Jenichen G, Denker K, Selleng S, Müllejans B, Greinacher A. Emergency transfusion of patients with unknown blood type with blood group O Rhesus D positive red blood cell concentrates: a prospective, single-centre, observational study. Lancet Haematol. 2017;4(5):e218–24. doi: 10.1016/S2352-3026(17)30051-0 28389344

[10] Dunbar NM, Yazer MH, OPTIMUS Study Investigators on behalf of the Biomedical Excellence for Safer Transfusion (BEST) Collaborative. O- product transfusion, inventory management, and utilization during shortage: the OPTIMUS study. Transfusion. 2018;58(6):1348–55. doi: 10.1111/trf.14547 29479703

[11] Virk MS, Lancaster D, Quach T, Lim A, Shu E, Belanger G, et al. Optimizing O-negative RBC utilization using a data-driven approach. Transfusion. 2020;60(4):739–46. doi: 10.1111/trf.15713 32077488

[12] Luyten U, Peeraer S, Pirlet C, Khaouch Y, Streel C, Deneys V. O-negative blood shortage management in a university hospital: Impact of transfusing RhD-positive red blood cells to RhD-negative patients. Transfus Clin Biol. 2023;30(4):402–9. doi: 10.1016/j.tracli.2023.06.007 37453488

[13] Reid ME, Lomas-Francis C, Olsson ML. The Blood Group Antigen FactsBook. 3rd ed. Elsevier. 2012.

[14] Cohn CS, Delaney M, Johnson ST, Katz LM, Schwartz J. Technical manual. 21st ed. AABB. 2023.

[15] American Red Cross. Facts about blood and blood types. Accessed 2023 November 9. https://www.redcrossblood.org/donate-blood/blood-types.html

[16] McAuliffe M, Oucho LA. World Migration Report 2024. Geneva, Switzerland: International Organization for Migration (IOM). 2024.

[17] Americas Blood Centers. U.S. Blood Donation Statistics and Public Messaging Guide. Americas Blood Centers. 2024.

[18] AABB. FAQs about Blood and Blood Donation. Accessed 2025 April 24. https://www.aabb.org/for-donors-patients/faqs-about-blood-and-blood-donation

[19] Garratty G, Glynn SA, McEntire R, Retrovirus Epidemiology Donor Study. ABO and Rh(D) phenotype frequencies of different racial/ethnic groups in the United States. Transfusion. 2004;44(5):703–6. doi: 10.1111/j.1537-2995.2004.03338.x 15104651

[20] American Red Cross. Blood Supply Statistics. Accessed 2025 April 24. https://www.redcrossblood.org/donate-blood/how-to-donate/how-blood-donations-help/blood-needs-blood-supply.html

[21] Shah MM, Spencer BR, James-Gist J, Haynes JM, Feldstein LR, Stramer SL, et al. Long-Term Symptoms Associated With SARS-CoV-2 Infection Among Blood Donors. JAMA Netw Open. 2024;7(4):e245611. doi: 10.1001/jamanetworkopen.2024.5611 38587842 PMC11002700

[22] US Census Bureau. Measuring Racial and Ethnic Diversity for the 2020 Census. Accessed 2025 August 21. https://www.census.gov/newsroom/blogs/random-samplings/2021/08/measuring-racial-ethnic-diversity-2020-census.html

[23] US Census Bureau. QuickFacts. Accessed 2023 November 9. https://www.census.gov/quickfacts/

[24] Jensen E, Jones N, Rabe M, Pratt B, Medina L, Orozco K, et al. 2020 U.S. Population More Racially and Ethnically Diverse Than Measured in 2010. 2021. Accessed 2025 May 1. https://www.census.gov/library/stories/2021/08/2020-united-states-population-more-racially-ethnically-diverse-than-2010.html

[25] Blakeslee L, Caplan Z, Meyer JA, Rabe MA, Roberts AW. Age and sex composition: 2020. U.S. Census Bureau. 2023. https://www.census.gov/library/publications.html

[26] Caplan Z, Rabe MA. The Older Population: 2020. U.S. Census Bureau. 2023. https://www.census.gov/library/publications.html

[27] Vyas DA, Eisenstein LG, Jones DS. Hidden in Plain Sight - Reconsidering the Use of Race Correction in Clinical Algorithms. N Engl J Med. 2020;383(9):874–82. doi: 10.1056/NEJMms2004740 32853499

[28] Malvik N, Leon J, Schlueter AJ, Wu C, Knudson CM. ABO-incompatible platelets are associated with increased transfusion reaction rates. Transfusion. 2020;60(2):285–93. doi: 10.1111/trf.15655 31912889 PMC7769037

[29] Gilstad CW, Conry-Cantilena K, Zarpak R, Eder AF. An outbreak of anaphylactic transfusion reactions to group B plasma and platelets and its possible relationship to Alpha-Gal syndrome. Transfusion. 2023;63(10):1997–2000. doi: 10.1111/trf.17521 37642435

[30] Miller MJ, Lee P, Lee BG, Flegel WA, West-Mitchell K, Conry-Cantilena K, et al. Consideration for alpha-gal syndrome in two critically ill persons with group O blood who received group B plasma. Transfusion. 2024;64(5):949–51. doi: 10.1111/trf.17811 38566573 PMC11104486

[31] Jones VM, Nakahara H, Choudhary SK, Dunbar NM, Szczepiorkowski ZM, Ratcliffe NR, et al. Immunologic investigation of an allergic transfusion reaction suspected due to alpha-gal syndrome. Transfusion. 2025;65(10):1989–95. doi: 10.1111/trf.18405 40938245

[32] Malard L, Beuscart C, de Chaisemartin L, de la Taille V, Delettre F, Humbrecht C, et al. Association between group B major ABO-incompatible platelet or plasma transfusion and severe allergic reactions: A nationwide haemovigilance analysis suggesting a role for α-gal syndrome. A Biomedical Excellence for Safer Transfusion Collaborative study. Vox Sang. 2026;121(1):81–6. doi: 10.1111/vox.70136 41161810

[33] Dunbar NM, Kaufman RM, Bary KS, Bellairs GRM, Cohn CS, Delettre F, et al. ABO-mismatched platelet and plasma transfusion practices and the potential for transfusion-related alpha-gal syndrome: The Biomedical Excellence for Safer Transfusion Collaborative Study. Transfusion. 2025;65(9):1621–9. doi: 10.1111/trf.18338 40643108

[34] Bougie DW, Reese SE, Birch RJ, Bookwalter DB, Mitchell PK, Roh D, et al. Associations between ABO non-identical platelet transfusions and patient outcomes-A multicenter retrospective analysis. Transfusion. 2023;63(5):960–72. doi: 10.1111/trf.17319 36994786 PMC10175171

[35] Cheng Z, Kong Y, Lin Y, Mi Z, Xiao L, Liu Z, et al. Transfusion outcomes and clinical safety of ABO-nonidentical platelets transfusion: A systematic review and meta-analysis. Transfus Apher Sci. 2024;63(4):103943. doi: 10.1016/j.transci.2024.103943 38820943

[36] Chousal JN, Sargolzaeiaval F, Huynh TR, Zhao M, Rodberg K, Kopko PM, et al. Hemolysis due to anti-IH in a patient with beta-thalassemia and Mycoplasma pneumoniae infection. Immunohematology. 2024;40(4):139–44. doi: 10.2478/immunohematology-2024-018 39740016

[37] Zeller MP, Barty R, Aandahl A, Apelseth TO, Callum J, Dunbar NM, et al. An international investigation into O red blood cell unit administration in hospitals: the GRoup O Utilization Patterns (GROUP) study. Transfusion. 2017;57(10):2329–37. doi: 10.1111/trf.14255 28840943

[38] Ilmakunnas M, Salmela K, Kivipuro T, Sareneva H, Sainio S. Use of O RhD-negative red blood cells: A nationwide, prospective audit. Vox Sang. 2022;117(11):1279–86. doi: 10.1111/vox.13357 36102215

[39] Gonzalez-Porras JR, Graciani IF, Perez-Simon JA, Martin-Sanchez J, Encinas C, Conde MP, et al. Prospective evaluation of a transfusion policy of D+ red blood cells into D- patients. Transfusion. 2008;48(7):1318–24. doi: 10.1111/j.1537-2995.2008.01700.x 18422846

[40] Ji Y, Luo G, Fu Y. Incidence of anti-D alloimmunization in D-negative individuals receiving D-positive red blood cell transfusion: A systematic review and meta-analysis. Vox Sang. 2022;117(5):633–40. doi: 10.1111/vox.13232 35014050

[41] U.S. Department of Health and Human Services. Find out if you can give blood. Accessed 2026 June 25. https://www.hhs.gov/givingequalsliving/giveblood/can-i-give

