# Supplementary material for: ABO and RhD blood group antigen frequencies in patients and blood donors: Implications for the U.S. blood supply
Source: PLoS One. 2026 Aug 25;21(8):e0356683. doi: 10.1371/journal.pone.0356683 (PMC13505927; doi:10.1371/journal.pone.0356683)
Supplement: Supplementary file 1 [file pone.0356683.s001.docx]

**Supporting information**

**Table S1. Characteristics of the five UC medical centers**

|  | **Davis** | **Irvine** | **Los Angeles** | **San Diego** | **San Francisco** |
| --- | --- | --- | --- | --- | --- |
| Number of hospital beds | 649 | 459 | 700 | 799 | 942 |
| Electronic health record | Epic | Epic | Epic | Epic | Epic |
| Laboratory information system | WellSky | Sunquest | SafeTrace Tx | SoftLab | Sunquest |
| Primary method for blood typing | Automated CAT | Automated solid-phase technique | Automated CAT | Automated CAT | Automated CAT |
| Approximate units transfused annually, all product types* (n) | 38,200 | 25,400 | 80,800 | 37,500 | 59,100 |

CAT—column agglutination technique

* Including red cell, plasma, platelet, and cryoprecipitate units
