# Supplementary material for: ABO and RhD blood group antigen frequencies in patients and blood donors: Implications for the U.S. blood supply
Source: PLoS One. 2026 Aug 25;21(8):e0356683. doi: 10.1371/journal.pone.0356683 (PMC13505927; doi:10.1371/journal.pone.0356683)
Supplement: Supplementary file 2 [file pone.0356683.s002.docx]

**Supporting information**

**Table S2. Harmonized terminology for self-reported race**

| **Source** | **Source Response Option (Verbatim)** | **Study Category** |
| --- | --- | --- |
| American Red Cross | African American | Black or African American |
| American Red Cross | Asian | Asian |
| American Red Cross | Caucasian | White |
| American Red Cross | Hispanic | Other |
| American Red Cross | Mix | Other |
| American Red Cross | Native American | American Indian or Alaska Native |
| American Red Cross | Other | Other |
| American Red Cross | Prefer not to answer | Not Reported/Unknown |
| UC Davis | American Indian or Alaska Native | American Indian or Alaska Native |
| UC Davis | Asian | Asian |
| UC Davis | Asked but no answer | Not Reported/Unknown |
| UC Davis | Black or African American | Black or African American |
| UC Davis | Native Hawaiian, Other Pacific Islander | Native Hawaiian or Pacific Islander |
| UC Davis | None of the above | Other |
| UC Davis | Unknown | Not Reported/Unknown |
| UC Davis | White | White |
| UC Irvine | American Indian or Alaska Native | American Indian or Alaska Native |
| UC Irvine | Asian | Asian |
| UC Irvine | Black or African American | Black or African American |
| UC Irvine | Native Hawaiian or Other Pacific Islander | Native Hawaiian or Pacific Islander |
| UC Irvine | Other Race or Mixed Race | Other |
| UC Irvine | Unknown (Patient cannot or refuses to declare race) | Not Reported/Unknown |
| UC Irvine | White | White |
| UC Los Angeles | American Indian or Alaska Native | American Indian or Alaska Native |
| UC Los Angeles | Asian | Asian |
| UC Los Angeles | Black or African American | Black or African American |
| UC Los Angeles | Multiple Races | Other |
| UC Los Angeles | Native Hawaiian or Other Pacific Islander | Native Hawaiian or Pacific Islander |
| UC Los Angeles | No Usable Values | Not Reported/Unknown |
| UC Los Angeles | White | White |
| UC San Diego | American Indian or Alaska Native | American Indian or Alaska Native |
| UC San Diego | Asian | Asian |
| UC San Diego | Black or African American | Black or African American |
| UC San Diego | Native Hawaiian or Other Pacific Islander | Native Hawaiian or Pacific Islander |
| UC San Diego | None of the Above | Other |
| UC San Diego | Not reported/Unknown | Not Reported/Unknown |
| UC San Diego | Other/Mixed | Other |
| UC San Diego | White | White |
| UC San Francisco | Asian | Asian |
| UC San Francisco | Black or African American | Black or African American |
| UC San Francisco | Declined/Unknown | Not Reported/Unknown |
| UC San Francisco | Native American or Alaska Native | American Indian or Alaska Native |
| UC San Francisco | None of the Above | Other |
| UC San Francisco | Other | Other |
| UC San Francisco | White | White |
| US Census 2020 | American Indian and Alaska Native | American Indian or Alaska Native |
| US Census 2020 | Asian | Asian |
| US Census 2020 | Black or African American | Black or African American |
| US Census 2020 | Native Hawaiian and Other Pacific Islander | Native Hawaiian or Pacific Islander |
| US Census 2020 | Other race or multiple races | Other |
| US Census 2020 | Some Other Race | Other |
| US Census 2020 | Two or More Races | Other |
| US Census 2020 | White | White |
