# Supplementary material for: ABO and RhD blood group antigen frequencies in patients and blood donors: Implications for the U.S. blood supply
Source: PLoS One. 2026 Aug 25;21(8):e0356683. doi: 10.1371/journal.pone.0356683 (PMC13505927; doi:10.1371/journal.pone.0356683)
Supplement: Supplementary file 3 [file pone.0356683.s003.docx]

**Supporting information**

**Table S3. Blood group antigen frequencies among California blood donors and US blood donors at the American Red Cross by race.***

|  | **O, RhD +** | **O, RhD -** | **A, RhD +** | **A, RhD -** | **B, RhD +** | **B, RhD -** | **AB, RhD +** | **AB, RhD -** |  | **Total** |
| --- | --- | --- | --- | --- | --- | --- | --- | --- | --- | --- |
| **California Donors** |  | |  | |  | |  | |  | 719,211 |
| American Indian or Alaska Native | 1,524 | 135 | 816 | 86 | 221 | 15 | 73 | 12 |  | 2,882 |
| Asian | 36,558 | 685 | 22,531 | 360 | 24,192 | 403 | 6,597 | 101 |  | 91,427 |
| Black or African American | 10,436 | 951 | 4,893 | 390 | 3,718 | 276 | 865 | 59 |  | 21,588 |
| Native Hawaiian or Pacific Islander | n/a | n/a | n/a | n/a | n/a | n/a | n/a | n/a |  | n/a |
| White | 128,387 | 32,187 | 104,510 | 21,380 | 30,222 | 6,173 | 11,808 | 2,409 |  | 337,076 |
| Other | 141,172 | 9,856 | 68,712 | 4,902 | 25,366 | 1,701 | 6,505 | 502 |  | 258,716 |
| Hispanic | 120,780 | 7,733 | 54,843 | 3,596 | 18,741 | 1,154 | 4,361 | 305 |  | 211,513 |
| Other race | 5,665 | 622 | 4,460 | 437 | 2,569 | 249 | 858 | 84 |  | 14,944 |
| Multiple races | 14,727 | 1,501 | 9,409 | 869 | 4,056 | 298 | 1,286 | 113 |  | 32,259 |
| Not Reported/Unknown | 3,303 | 445 | 2,148 | 294 | 958 | 73 | 265 | 36 |  | 7,522 |
|  |  |  |  |  |  |  |  |  |  |  |
| **US Donors** |  |  |  |  |  |  |  |  |  | 7,449,831 |
| American Indian or Alaska Native | 14,943 | 1,929 | 9,026 | 1,126 | 2,299 | 286 | 746 | 98 |  | 30,453 |
| Asian | 113,162 | 3,829 | 69,840 | 2,056 | 77,771 | 2,370 | 20,511 | 620 |  | 290,159 |
| Black or African American | 164,701 | 13,678 | 76,122 | 5,677 | 60,299 | 4,487 | 13,322 | 1,034 |  | 339,320 |
| Hispanic | 288,382 | 21,786 | 133,666 | 10,279 | 44,608 | 3,313 | 10,408 | 882 |  | 513,324 |
| Native Hawaiian or Pacific Islander | n/a | n/a | n/a | n/a | n/a | n/a | n/a | n/a |  | n/a |
| White | 2,308,455 | 601,733 | 1,887,095 | 413,250 | 500,475 | 111,974 | 193,579 | 41,350 |  | 6,057,911 |
| Other | 369,682 | 31,234 | 189,232 | 15,949 | 70,940 | 5,743 | 18,693 | 1,667 |  | 703,140 |
| Hispanic | 288,382 | 21,786 | 133,666 | 10,279 | 44,608 | 3,313 | 10,408 | 882 |  | 513,324 |
| Other race | 22,805 | 2,735 | 17,064 | 1,840 | 9,602 | 924 | 3,047 | 312 |  | 58,329 |
| Multiple races | 58,495 | 6,713 | 38,502 | 3,830 | 16,730 | 1,506 | 5,238 | 473 |  | 131,487 |
| Not Reported/Unknown | 11,741 | 2,083 | 8,559 | 1,451 | 3,315 | 419 | 1,112 | 168 |  | 28,848 |

* Race is reported using OMB terminology. Actual terminology used by the American Red Cross: Native American, Asian, African American, Hispanic, Caucasian, Other, Mix, Prefer not to answer. Native Hawaiian or Pacific Islander is not used by the American Red Cross.
